# Ameliorative Effect of Citropten Isolated from *Citrus* *aurantifolia* Peel Extract as a Modulator of T Cell and Intestinal Epithelial Cell Activity in DSS-Induced Colitis

**DOI:** 10.3390/molecules27144633

**Published:** 2022-07-20

**Authors:** Hyun-Su Lee, Eun-Nam Kim, Gil-Saeng Jeong

**Affiliations:** 1 Department of Physiology, School of Medicine, Daegu Catholic University, Daegu 42472, Korea; lhs6858@cu.ac.kr; 2 College of Pharmacy, Chungnam National University, Daejeon 34134, Korea; enkim@cnu.ac.kr

**Keywords:** citropten, colitis, T cell activation, intestinal cell activation, anti-inflammation, MAPK

## Abstract

Citropten is a coumarin that is mainly found in fruits of Rutaceae trees, but its anti-inflammatory activities in colitis is still unknown. In this study, we investigated its attenuating effect of citropten isolated from *Citrus aurantifolia* extract on DSS-induced colitis through the modulation of the activity of T cells and intestinal epithelial cells. We found that pre-treatment with citropten downregulates the activity of T cells and intestinal epithelial cells without a negative effect on the viability of Jurkat and HT-29 cells. The results from the Western blot analysis revealed that pre-treatment with citropten reduces the NFκB and MAPK signaling pathway in activated T cells and intestinal epithelial cells. We elucidated that the oral administration of citropten alleviates the colonic inflammation and activity of effector T cells in DSS-induced colitis by measuring changes in body weight, histological scoring from H&E-stained sections, mRNA levels of pro-inflammatory cytokines and the phosphorylation level of the MAPK signaling pathway.

## 1. Introduction

Colitis has been investigated as an inflammatory disorder provoked by several factors including infection, allergic reactions and inflammatory bowel disease (IBD) [1]. IBD is a chronic disease of the digestive tract, mainly ulcerative colitis (UC) and Crohn’s disease (CD), characterized by the chronic and spontaneous recurrence of inflammation. The symptoms of IBD include abdominal pain, cramping and diarrhea with blood in the stool [2]. The accurate etiology of IBD is still unknown, but accumulated evidence shows that it is correlated with uncontrolled excessive immune responses [3,4]. It has been elucidated that the uncontrolled activation of mucosal T cells causes mucosal damage during IBD which results in functional changes as well as tissue destructions [5]. Since intestinal epithelial cells play a pivotal role as a first barrier that defends against pathogens, it is important to maintain immunological homeostasis, including the activity of intestinal epithelial cells, during IBD [6]. Even though the inhibition or interruption of immunopathogenic responses including T cells and intestinal epithelial cells can be a potential therapeutic strategy, this still needs to be properly proven.

The engagement of T cell receptor (TCR) and major histocompatibility complex (MHC) molecules with antigenic peptides is one of the critical events that provokes a T-cell-mediated immune response [7]. CD28 is also involved as a costimulatory molecule in T cell activation which leads to the activation of the nuclear kappa-light-chain-enhancer of activated B cells (NFκB) pathway through the NFκB binding site on the CD28 response element (CD28RE) [8]. It has been well studied as one of most important transcription factors that translocates into the nucleus in an active state [9]. It has been also reported that the NFκB pathway in intestinal epithelial cells plays a crucial role in the maintenance of immune homeostasis during inflammatory colitis [10,11]. Nevertheless, considering the fact that the activation of the NFκB pathway is involved in disease activity in IBD patients, efforts to develop treatments targeting the NFκB pathway remain insufficient.

Citropten (5,7-dimethoxycoumarin or limettin, C_11_H_10_O_4_) is one of coumarin derivatives that possesses a variety of biological properties including antioxidant and anti-cancer activities [12,13]. Several studies in the literature have reported that it is isolated from *Citrus limon*, *Carica papaya* and *Citrus bergamia* [14,15,16]. It has been also elucidated that it has diverse biological functions, including an anti-proliferating effect on B16 melanoma cells, an inhibitory effect on MAPK in carcinoma tissues and a preventive effect on chronic-depression-induced mild stress in rats [17,18,19]. Though various activities of citropten have been well established, little is known as to whether citropten has an anti-inflammatory effect on inflammatory colitis.

In the current study, we investigated the ameliorative effect of citropten isolated from *Citrus aurantifolia* extract on inflammatory colitis using a DSS-induced colitis animal model. The modulation of activities of T cells and intestinal epithelial cells through the suppression of the nuclear translocation of p65 in the NFκB pathway and the phosphorylation of MAPK signaling molecules through pre-treatment with citropten was presented as the underlying mechanism.

## 2. Results

### 2.1. Isolation of Citropten from C. aurantifolia Peel Extract and Its Chemical Structure

**Figure 1:**
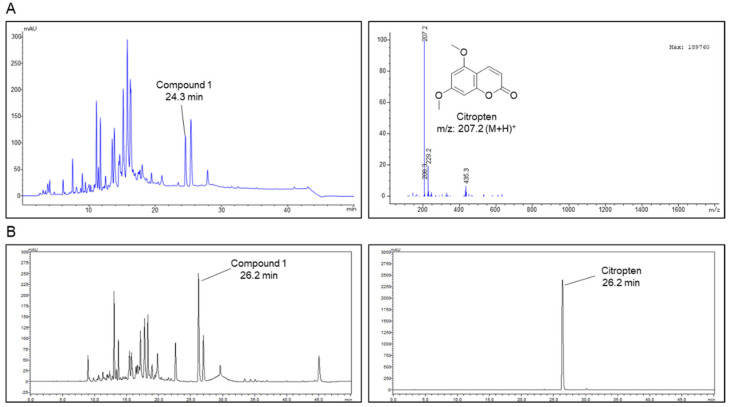
Isolation of citropten from *C. aurantifolia* peel extract and its chemical structure. (**A**) High-performance liquid chromatography (HPLC)-mass spectrum of *C. aurantifolia* peel 70% EtOH extract, and ESI-MS spectra of the [M + H]^+^ ion of citropten. (**B**) Purity evaluation of isolated citropten using HPLC-DAD (330 nm).

Liquid chromatography–mass spectrometry analysis was performed on the 70% EtOH extract of *C. aurantifolia* peel, and the MS spectrum for compound **1** detected at 26.2 min was obtained. Compound **1** isolated in the positive ion mode of MS spectrum showed a mass to charge ratio of 207.2 (Figure 1A). In addition, the purity of the isolated citropten was evaluated using HPLC-DAD, and the purity of citropten was confirmed to be about 98.65% (Figure 1B).

### 2.2. Citropten Has No Negative Effect on the Viability of Jurkat and HT-29 Cells

**Figure 2:**
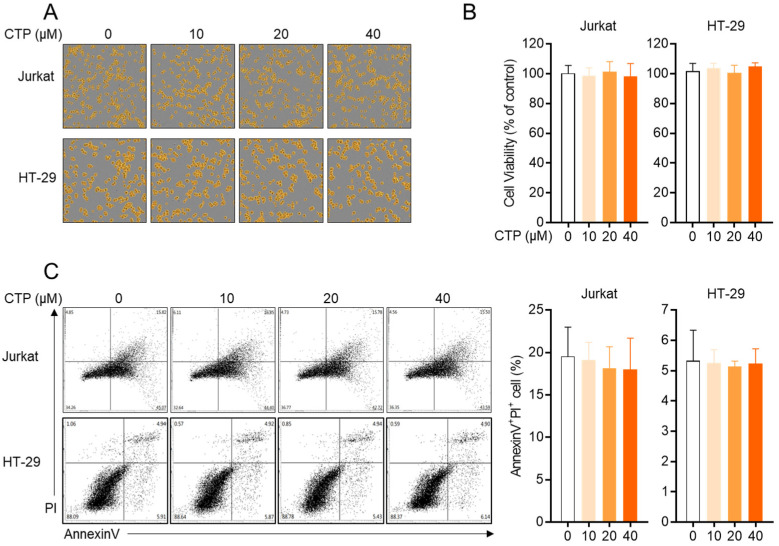
Citropten has no negative effect on the viability of Jurkat and HT-29 cells. (**A**,**B**) Jurkat cells (1 × 10^4^/well) and HT-29 cells (1 × 10^4^/well) were treated with indicated concentration (0 to 40 μM) of citropten for 24 h; then, cells were marked in orange using IncuCyte imaging system (**A**). Cell viability was determined by performing MTT assay (**B**). Cell viability was presented in % of control (0 μM). (**C**) Jurkat cells (5 × 10^5^/well) and HT-29 cells (5 × 10^5^/well) were treated with indicated concentrations (0 to 40 μM) of citropten for 24 h; then, AnnexinV^+^PI^+^ double-positive cells were detected by performing AnnexinV/PI apoptosis assay. Results are expressed as mean ± SD of three independent experiments.

Since the cytotoxicity of compounds has been mainly reported as an underlying mechanism of the inhibitory effect on the activity, first, we explored whether citropten leads to cytotoxicity in T cells and epithelial intestinal cells. For the in vitro assay, Jurkat T cells and HT-29 cells were used in the present study. Figure 2A shows that the confluency of both cells is not affected by treatment with citropten up to 40 μM. To estimate the cellular viability in the presence of citropten, an MTT viability assay was performed using Jurkat cells and HT-29 cells. Comparable viability was revealed in Jurkat and HT-29 cells incubated with citropten for 24 h up to 40 μM (Figure 2B). To confirm whether treatment with citropten induces apoptosis-related cell death in Jurkat and HT-29 cells, an AnnexinV/PI apoptosis assay was performed. The population of AnnexinV/PI double-positive cells was measured via flow cytometry. Figure 2C shows that treatment with citropten up to 40 μM did not change the percentage of Jurkat and HT-29 cells expressing AnnexinV and PI. These results suggest that citropten treatment up to 40 μM does not have a negative effect on the viability of Jurkat and HT-29 cells.

### 2.3. Activity of Jurkat T Cells Is Downregulated by Pre-Treatment with Citropten

**Figure 3:**
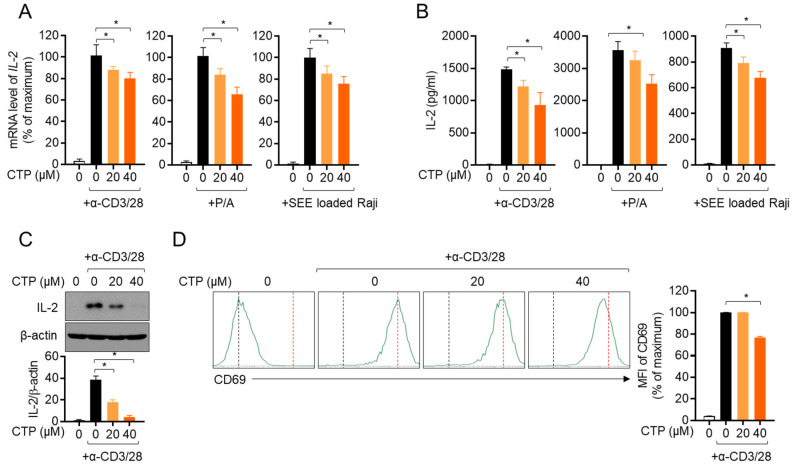
Activity of Jurkat T cells is downregulated by pre-treatment with citropten. (**A**,**B**) Jurkat T cells (5 × 10^5^/well) were pre-treated with the indicated concentration (0 to 40 μM) of citropten for 1 h and stimulated with anti-CD3 antibodies (20 μg/mL) and anti-CD28 antibodies (7 μg/mL, left), PMA (100 nM) and A23187- (1 μM, middle) or SEE-loaded Raji B cells (right) for 6 h (**A**) or 24 h (**B**). mRNA level of IL-2 was detected via real-time quantitative PCR (**A**), and released IL-2 was measured using ELISA (**B**). (**C**) Jurkat T cells (1 × 10^6^/well) were pre-treated with the indicated concentration (0 to 40 μM) of citropten for 1 h and stimulated with anti-CD3 antibodies (20 μg/mL) and anti-CD28 antibodies (7 μg/mL) for 6 h. Cells were harvested and lysed in RIPA buffer. Produced IL-2 was detected via Western blot analysis. Detected IL-2 was normalized with the intensity of β-actin and is shown in bar graph below. (**D**) Pre-treated Jurkat cells (5 × 10^5^) with 40 μM of citropten were stimulated with anti-CD3 antibodies (20 μg/mL) and anti-CD28 antibodies (7 μg/mL) for 16 h. After stimulation, cells were stained with anti-CD69 antibodies conjugated with APC and acquired for detection of fluorescence via flow cytometry. Each plot is presented, and mean fluorescence intensity is shown in bar graph. Results are expressed as mean ± SD of three independent experiments (*, *p* < 0.05).

Since the activation of T cells in the inflammatory response plays a pivotal role in the pathogenesis of colitis, we investigated whether citropten affects T cell activity in vitro. Three in vitro models of stimulating T cells were used in the present study, including TCR-mediated stimulation using anti-CD3 and anti-CD28 antibodies, treatment with PMA and A23187 and a co-culture system with superantigen-loaded Raji B cells. Figure 3A shows that pre-treatment with citropten inhibited the mRNA level of IL-2 from activated Jurkat T cells in a dose-dependent manner. It was confirmed that released IL-2 from activated T cells is suppressed by citropten (Figure 3B). To examine whether citropten blocks IL-2 production from stimulated T cells, cytosolic IL-2 was detected using a Western blot assay. Figure 3C shows that pre-treatment with citropten downregulated IL-2 production in activated T cells. The expression of CD69, which is one of the specific markers of T cell activation, on the surface of activated T cells was monitored using flow cytometry. As shown in Figure 3D, the intensity of CD69 was significantly decreased by pre-treatment with citropten. These results suggest that citropten effectively abrogates T cell activation, including IL-2 production as well as CD69 expression, from activated T cells.

### 2.4. Citropten Suppresses the Production of Inflammatory Cytokines in Activated HT-29 Cells

**Figure 4:**
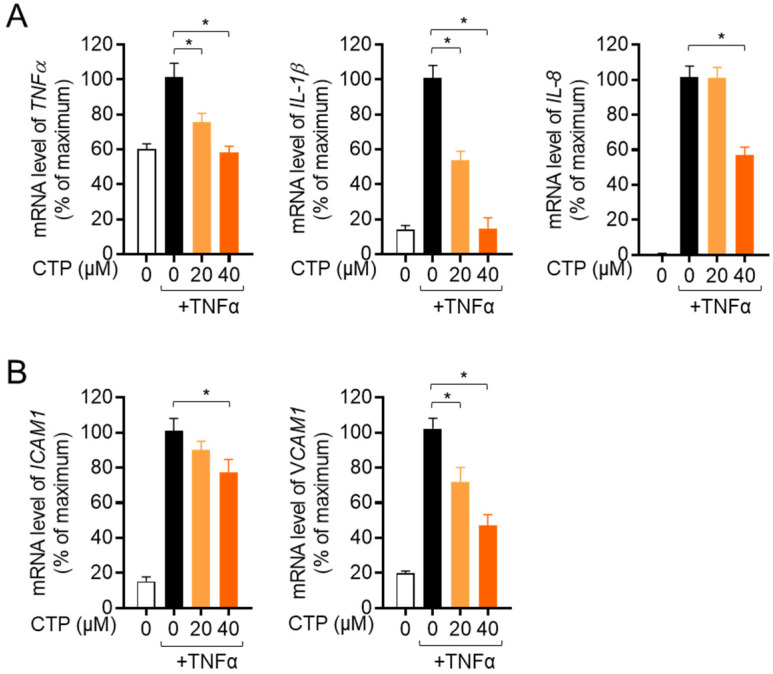
Citropten suppresses the production of inflammatory cytokines in activated HT-29 cells. (**A**, **B**) HT-29 cells were pre-treated with the indicated concentration (0 to 40 μM) for 1 h and stimulated with recombinant TNFα (10 ng/mL) for 6 h. mRNA levels of TNFα, IL-1β and IL-8 (**A**) or ICAM1 and VCAM1 (**B**) were measured via real-time quantitative PCR analysis. The value was presented in % of maximum by normalization with GAPDH. Results are expressed as mean ± SD of three independent experiments (*, *p* < 0.05).

We further investigated whether citropten has a modulatory effect on the production of inflammatory cytokines from activated HT-29 by treatment with recombinant TNFα. The mRNA levels of pro-inflammatory cytokines including TNFα, IL-1β and IL-8 were explored in the presence or absence of pre-treatment with citropten of HT-29 cells via real-time quantitative PCR. Figure 4A shows that pre-treatment with dose-dependent citropten reduced the mRNA levels of pro-inflammatory cytokines. To elucidate whether pre-treatment with citropten modulated the expression of surface molecules on activated cells by recombinant TNFα, we checked the mRNA levels of ICAM1 and VCAM1 via real-time quantitative PCR. A downregulated mRNA level of ICAM1 and VCAM1 was observed on activated HT-29 cells pre-treated with citropten in a dose-dependent manner. These data suggest that pre-treatment with citropten effectively regulates the activity of HT-29 cells in terms of the production of pro-inflammatory cytokines and the expression of surface molecules.

### 2.5. Pre-Treatment with Citropten Reduces NFκB and MAPK Signaling Pathway in Activated Jurkat and HT-29

**Figure 5:**
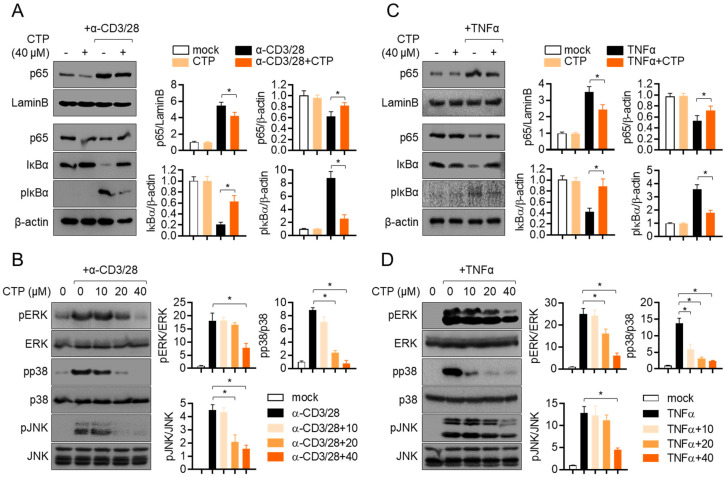
Pre-treatment with citropten reduces NFκB and MAPK signaling pathway in activated Jurkat and HT-29. (**A**,**B**) Jurkat cells were pre-treated with 40 μM (**A**) or the indicated concentration (0 to 40 μM, (**B**)) of citropten and were stimulated with anti-CD3 antibodies (20 μg/mL) and anti-CD28 antibodies (7 μg/mL) for 1 h (**A**) or 30 min (**B**). For separation of nucleic extract (**A**), cells were lysed by using NE-PER kit. Nuclear transported p65 was detected in nuclear extract and cytosolic extract. Degraded and phosphorylated IκBα were detected in cytosolic extract. Detected proteins were normalized with the intensity of loading control proteins (LaminB for nuclear extract and β-actin for cytosolic extract). For the detection of phosphorylated level (**B**), harvested cells were lysed in RIPA buffer. Phosphorylated and total protein of ERK, p38 and JNK were determined via Western blot analysis. Phosphorylated levels were normalized with the intensity of total proteins and are presented in bar graphs. (**C**,**D**) HT-29 cells were pre-treated with 40 μM (**C**) or the indicated concentration (0 to 40 μM, (**D**)) of citropten and were stimulated with recombinant TNFα (10 ng/mL) for 1 h (**C**) or 30 min (**D**). For separation of nucleic extract (**C**), cells were lysed by using NE-PER kit. Nuclear transported p65 was detected in nuclear extract and cytosolic extract, respectively. Degraded and phosphorylated IκBα were detected in cytosolic extract. Detected proteins were normalized with the intensity of loading control proteins (LaminB for nuclear extract and β-actin for cytosolic extract). For the detection of phosphorylated level (**D**), harvested cells were lysed in RIPA buffer. Phosphorylated and total protein of ERK, p38 and JNK were determined via Western blot analysis. Phosphorylated levels were normalized with the intensity of total proteins and are presented in bar graphs. Results are expressed as mean ± SD of three independent experiments (*, *p* < 0.05).

It has been well reported that NFκB is a one of the main transcription factors that is involved in T cell activation. To investigate whether pre-treatment with citropten affects to the NFκB signaling pathway in TCR-mediated activation, the nuclear translation of p65 was assessed via Western blot analysis. Activated T cells showed the increased translocation of nuclear p65, but pre-treatment with citropten slightly reduced it, and simultaneously, the remaining p65 in cytosol was increased in activated T cells pre-treated with citropten (Figure 5A). To explore how the activity of IκBα is affected by pre-treatment with citropten in activated T cells, the degradation and phosphorylation of IκBα was examined. Figure 5A shows that the enhanced degradation and phosphorylation of IκBα in activated T cells were significantly suppressed by pre-treatment with citropten. We further examined whether the MAPK signaling pathway is affected by pre-treatment with citropten in activated T cells. Figure 5B shows that the phosphorylation levels of ERK, p38 and JNK were augmented by TCR-mediated stimulation, but pre-treatment with citropten inhibited them in a dose-dependent manner. We aimed to confirm whether the modulatory effect of citropten through the NFκB and MAPK signaling pathway is also shown in activated HT-29 cells by TNFα. Figure 5C,D reveals that citropten pre-treatment had a negative effect on the NFκB and MAPK signaling pathway in activated HT-29 cells. These data suggest that pre-treatment with citropten suppresses the activity of T cells and epithelial intestinal cells through the NFκB and MAPK signaling pathway in vitro.

### 2.6. Oral Administration of Citropten Attenuates DSS-Induced Colitis in Mice Model

**Figure 6:**
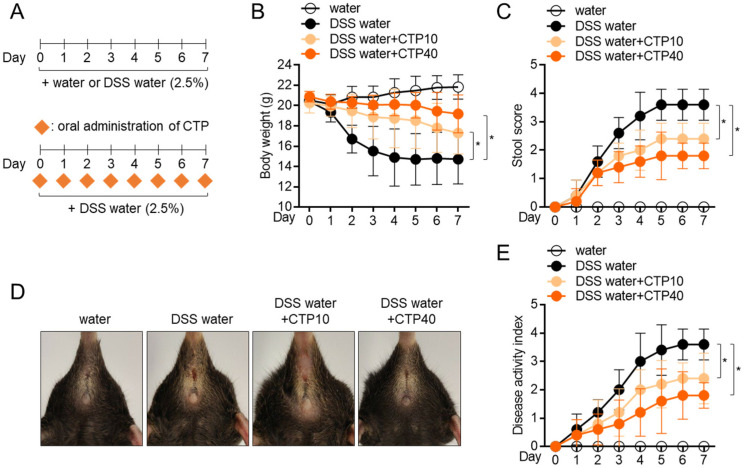
Oral administration of citropten attenuates DSS-induced colitis in mice model. (**A**) Experimental scheme of IBD induction. (**B**) Transition of body weight during 7 days of IBD induction. (**C**) Transition of stool score during 7 days of IBD induction. (**D**) Representative pictures of mice anus on day 7 post-induction. (**E**) Transition of disease activity index during 7 days of IBD induction. Data are expressed as mean ± SD (*n* = 5/group) (*, *p* < 0.05). Water: mouse group fed with fresh water, DSS water: mouse group fed with water containing 2.5% DSS, DSS water + CTP10; mouse group fed with water containing 2.5% DSS and orally administered with 10 mg/kg citropten, DSS water + CTP40; mouse group fed with water containing 2.5% DSS and orally administered with 40 mg/kg citropten.

To explore whether citropten has an ameliorative effect on inflammatory colitis in vivo, a DSS-induced colitis model was used. An inflammatory colitis model was used by feeding mice water containing 2.5% DSS for 7 days in the presence of the daily oral administration of citropten. To obtain more accurate results, two doses of citropten (10 mg/kg and 40 mg/kg) were used in animal model experiments (Figure 6A). Feeding water containing 2.5% DSS significantly reduced the body weight of mice due to severe inflammatory responses, but the oral administration of citropten protected weight loss caused by the feeding of DSS-containing water (Figure 6B). To measure how severely the colitis lesion progressed, a daily stool score was measured according to the pathological criteria. Figure 6C reveals that mice with colitis who underwent the oral administration of citropten exhibited a downregulated stool score. Pictures of the anuses of mice which were taken on day 7 showed improved manifestation in the mouse group with the oral administration of citropten (Figure 6D). The assessed disease activity index (DAI) also confirmed that the oral administration of citropten decreased various disease levels in the colitis model (Figure 6E). These data suggest that the oral administration of citropten ameliorates the manifestations of inflammatory colitis.

### 2.7. Oral Administration of Citropten Alleviates the Colonic Inflammation in DSS-Induced Colitis Model

**Figure 7:**
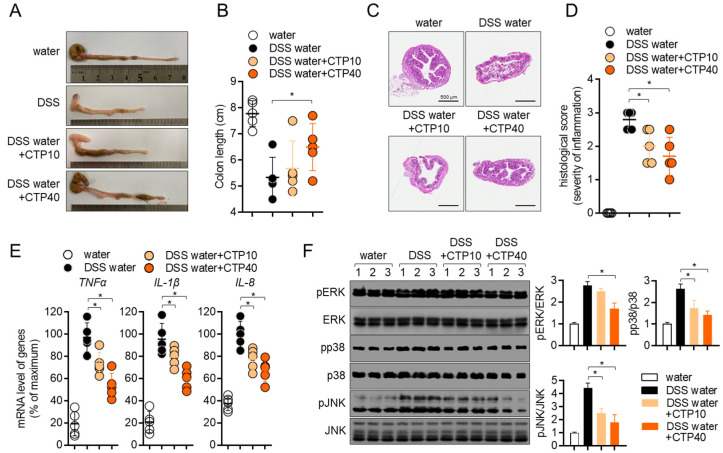
Oral application of citropten alleviates the colonic inflammation in DSS-induced colitis. (**A**) Representative photographs of colons from each group of mice at day 7. (**B**) Length of colons from (**A**). (**C**) Colons were removed on day 7 post-IBD induction, and sections were stained with H&E. Bars, 500 μm. (**D**) Histological scores of sections staining with H&E from (**C**). (**E**) mRNA levels of pro-inflammatory cytokines in colon tissues from each group of mice were measured via real-time quantitative PCR. The levels were normalized with GAPDH and presented in % of maximum. (**F**) Phosphorylated levels of ERK, p38 and JNK were detected via Western blot analysis on lysed colon tissues. Phosphorylated levels were normalized with the intensity of total proteins and are presented in bar graphs. Data are presented as mean ± SD (*n* = 5/group) (*, *p* < 0.05). Water: mouse group fed with fresh water, DSS water: mouse group fed with water containing 2.5% DSS, DSS water + CTP10: mouse group fed with water containing 2.5% DSS and orally administered with 10 mg/kg citropten, DSS water + CTP40: mouse group fed with water containing 2.5% DSS and orally administered with 40 mg/kg citropten.

To elucidate whether the oral administration of citropten leads to alteration in colons, colons tissues were removed, and the colonic length was measured. Figure 7A,B shows that colons from mice fed with 2.5% DSS water were shrunken in length due to inflammation, but the oral administration of citropten prevented the inflammatory shrinkage in a dose-dependent manner. The results obtained from tissue section staining with H&E revealed that the collapse of intestinal structures was restored by the oral administration of citropten (Figure 7C). In particular, the infiltration of inflammatory cells, the epithelial damages and crypt lesions were improved in a dose-dependent manner. The measurement of histological scores from obtained H&E tissue staining also confirmed that the oral administration of citropten ameliorated the severity of inflammation in colon tissue (Figure 7D). The mRNA levels of TNFα, IL-1β and IL-8 were determined using a real-time quantitative PCR to confirm whether the oral administration of citropten decreased to produce pro-inflammatory cytokines on colonic tissues. Figure 7E shows that elevated mRNA levels of TNFα, IL-1β and IL-8 were reduced by the oral administration of citropten in a dose-dependent manner. To prove the connection with the in vitro results and how the oral administration of citropten suppresses inflammatory responses in the colitis model, a Western blot analysis was performed using colonic tissues. Figure 7F shows that the MAPK signaling pathway, including the phosphorylation of ERK, p38 and JNK, was slightly affected by the oral administration of citropten. These data suggest that the oral administration of citropten modulated the inflammatory responses on colonic tissue in the colitis model.

### 2.8. Oral Treatment with Citropten Ameliorates T Cell Activity in DSS-Induced Colitis Model

**Figure 8:**
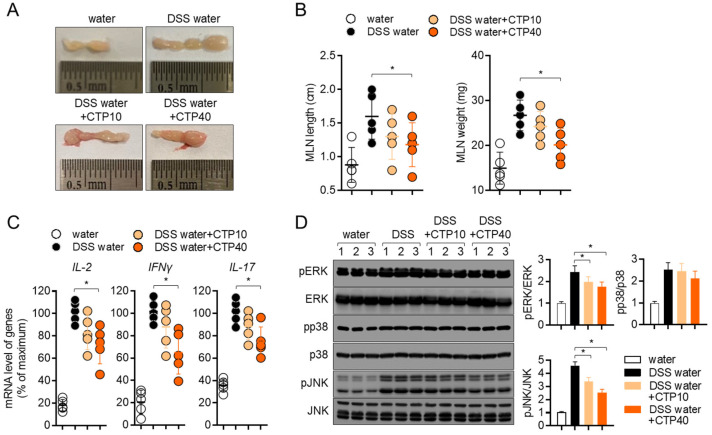
Oral treatment with citropten ameliorates T cell activity in DSS-induced colitis model. (**A**) Representative photographs of mesenteric lymph nodes from each group of mice at day 7. (**B**) Length and weight of mesenteric lymph nodes from (**A**). (**C**) mRNA levels of effector cytokines in mesenteric lymph nodes from each group of mice were measured via real-time quantitative PCR. The levels were normalized with *GAPDH* and presented in % of maximum. (**D**) Phosphorylated levels of ERK, p38 and JNK were detected via Western blot analysis on lysed lymph nodes. Phosphorylated levels were normalized with the intensity of total proteins and are presented in bar graphs. Data are presented as mean ± SD (*n* = 5/group) (*, *p* < 0.05). Water: mouse group fed with fresh water, DSS water: mouse group fed with water containing 2.5% DSS, DSS water + CTP10: mouse group fed with water containing 2.5% DSS and orally administered with 10 mg/kg citropten, DSS water + CTP40: mouse group fed with water containing 2.5% DSS and orally administered with 40 mg/kg citropten.

Since the activity of T cells has been investigated as a major player in colonic inflammation, we collected information regarding mesenteric lymph nodes at day 7 post-induction to compare the T cell activation in different groups. Figure 8A,B shows elevations in the length and weight of mesenteric lymph nodes, but the oral administration of citropten decreased them. To explore whether the oral application of citropten suppressed the activity of effector T cells in the colitis model, the mRNA levels of IL-2, IFNγ and IL-17 were measured via real-time quantitative PCR. Increased mRNA levels of IL-2, IFNγ and IL-17 in colons from DSS mice were significantly decreased via the oral administration of citropten in vivo. The phosphorylation level of ERK, p38 and JNK was also confirmed via Western blot analysis to find the relationship between in vitro results and in vivo manifestations. Figure 8D shows that ERK and JNK phosphorylation were slightly affected by the oral administration of citropten on mesenteric lymph nodes. These data suggest that the oral administration of citropten attenuates the activity of effector T cells in DSS-induced colitis in vivo.

## 3. Discussion

In the current study, we found that citropten isolated from *Poncirus trifoliate* extract attenuates the manifestations of DSS-induced colitis in vivo. We showed that pre-treatment with citropten effectively modulates the activity of T cells and intestinal epithelial cells through the NFκB and MAPK signaling pathway in vitro without cytotoxicity. These findings suggest that citropten has promising potential as an ingredient in therapeutic drugs for inflammatory colitis.

Traditionally, citrus plants have been considered to have abundant flavonoids and coumarins which show beneficial bioactivities such as antioxidant and anti-inflammation [20]. *C. aurantifolia* has been widely used in edible fruits that have unique flavors, and it contains a lot of vitamin C [21]. In particular, even though several reports have determined that vitamin C itself exerts the therapeutic effect on colitis, the protective effect of citropten on inflammatory colitis still elusive [22,23]. Our findings have scientific significance considering the traditional use of *C. aurantifolia*, which we used to isolate citropten for this study.

Since effector cytokines from activated T cells including IL-2 have been determined to be involved in endocrine, paracrine or autocrine proliferation in soluble forms [24], the amount of released IL-2 after stimulation is important. Several studies in the literature have elucidated that produced cytokines undergo a secretion process that is tightly controlled by vesicle trafficking molecules including the VAMPs family or SNAP molecules [25,26]. To understand whether the inhibitory effect of citropten on T cell activation is associated with the vesicle trafficking of cytokine release, we detected the cytosolic IL-2 that is produced after TCR-mediated stimulation. Figure 3C reveals that the modulatory effect of citropten on T cell activity is through an intrinsic mechanism including the downregulation of signaling pathways. The result that shows the expression of CD69, which is the marker of T cell activity on the surface, also supports the concept that citropten may induce intrinsic suppression on T cell activation.

It has been shown that lymphocytes including T cells and B cells migrate into mesenteric lymph nodes to encounter antigens, be primed and proliferate during inflammatory colitis [27]. As the number of cells migrating into mesenteric lymph nodes during colitis increased, the enhanced size and weight of mesenteric lymph nodes were observed [28]. Lymphadenopathy has been defined as the swelling of localized lymph nodes during inflammatory responses including infections or allergic responses [29]. In the results obtained from the present animal experiment, changes in the length and weight of mesenteric lymph nodes were found; the oral administration of citropten significantly reduced the length and weight of mesenteric lymph nodes (Figure 8B). These results suggest that the oral administration of citropten ameliorates the manifestation of lymphadenopathy by the modulation of inflammatory responses during DSS-induced colitis.

The NFκB signaling pathway is one of the most critical transcription factors in T cell activation and differentiation. It has been elucidated to be involved in polarization into Th1 via the induction of the required cytokines, including IL-12 [30]. Several reports have revealed that NFκB plays a critical role in Th17 differentiation from naïve T cells by positively affecting the gene expression of RORγt, which is the master transcription factor of Th17 differentiation [31,32]. In the current study, we showed that pre-treatment with citropten controls T cell activation through the NFκB signaling pathway in vitro (Figure 5A). We also determined that the mRNA levels of IFNγ and IL-17 on mesenteric lymph nodes were regulated by the oral administration of citropten in a DSS-induced colitis model (Figure 8C). These findings suggest that the oral administration of citropten may have an influence on T cell differentiation into Th1 and Th17 effector T cells, which are the most important subunits in the pathogenesis of inflammatory colitis through the modulation of the NFκB signaling pathway.

It was shown that MAPK involves several inflammatory disorders, including colitis, after it was discovered more than 25 years ago [33]. Among MAPK, the ERK1/2 pathway, the most evaluated subunit in inflammatory responses, has been determined to play a critical role in the progression and development of IBD and the generation of pro-inflammatory cytokines such as IL-1 and IL-21 [34,35]. Results from inhibitor studies of p38 have shown that the phosphorylation of p38 is dramatically enhanced in IBD tissues [36,37]. In addition, several publications have demonstrated that an elevated phosphorylation level of JNK was detected in IBD patients, and results from an inhibitor assay of JNK have revealed that the expression of proinflammatory cytokines was significantly downregulated in an inhibitor-administrated group in an animal model [37,38,39]. In the present study, we found that the phosphorylation levels of ERK, p38 and JNK were remarkably reduced in the colon tissues of the DSS group with the oral administration of citropten (Figure 7F). These results are highly relevant to in vitro data showing the modulatory effect of citropten on epithelial intestinal cells’ activation (Figure 5D).

ICAM1 and VCAM1 have been studied as adhesion molecules; their expressions are upregulated on intestinal epithelial cells in inflammatory colitis patients [40,41]. Since enhanced expressions of ICAM and VCAM1 play a critical role in the transmigration of leukocytes into the lesion of inflammatory colitis, several studies have shown their possibility as therapeutic targets of inflammatory colitis by blocking the activity of ICAM1 and VCAM1 [42,43]. These findings suggest that the transmigration of leukocytes and the interaction between inflammatory leukocytes and intestinal epithelial cells are pivotal for the development of inflammatory colitis. In the present study, we elucidated that pre-treatment with citropten mitigates the mRNA levels of ICAM1 and VCAM1 on activated intestinal epithelial cells via TNFα treatment (Figure 4B). In addition, we also revealed that the expression of IL-8 is downregulated by pre-treatment with citropten, which acts as a chemoattractant for leukocytes in colitis pathogenesis. The regulatory effects of citropten on the expression of ICAM1, VCAM1 and IL-8 can be applied to the development of new drugs for inflammatory colitis.

## 4. Materials and Methods

### 4.1. Cell Culture

Jurkat T cells (KCLB number: 40152) and HT-29 cells (KCLB number: 30038) were purchased from the Korean Cell Line Bank (Seoul, Korea), and Raji B (ATCC cat#. CCL-86) cells were obtained from ATCC (Manassas, VA, USA). Cells were cultured in RPMI (Jurkat cells and Raji cells) or DMEM (HT-29 cells) medium (Welgene, Gyeongsan, Korea) supplemented with penicillin G (100 units/mL), streptomycin (100 μg/mL), 10% fetal bovine serum (FBS) and l-glutamine (2 mM). Both cell lines were maintained within 10 passages and grown at 37 °C in a humidified incubator containing 5% CO_2_ and 95% air.

### 4.2. Animals

Six- to eight-week-old C57BL/6J female mice were purchased from Samtako Bio (Osan, Korea) and housed under specific pathogen-free (SPF) conditions. All experiments were approved by the Animal Care and Use Committee of the College of Pharmacy, Keimyung University (approval number: KM2020-004).

### 4.3. Plant Material

*C. aurantifolia* was purchased at the Daegu Yangnyeong Herbal Medicine Market in 2020, and a plant voucher specimen (KMU-2020-04-08) was deposited at Keimyung University College of Pharmacy.

### 4.4. Isolation of Citropten from C. aurantifolia Peel Extract

The *C. aurantifolia* the peels were manually separated, the pulp was deseeded and the separated peel was dried. The dried *C. aurantifolia* peels (265.0 g) were extracted with 70% EtOH (1 L) at room temperature for 1 day, and extraction was performed at a temperature of 60 degrees for 2 h. The alcoholic extract was evaporated in vacuo to yield a residue (41.2 g) and was partitioned with *n*-hexane, EtOAc, and *n*-butanol successively. Among them, the n-hexane fraction (16.00 g, Fr.1) was partitioned with silica gel column chromatography with a gradient elution of hexane–EtOAc (0–100 hexane/EtOAc *v*/*v*%), affording 14 fractions (Fr. 1–1~14). A white precipitate of Fr. 1–8 (87.6 mg) in the separated fraction was recrystallized in chloroform to obtain compound **1** (11.6 mg). The ^1^H and ^13^C nuclear magnetic resonance and liquid chromatography–mass spectrometry analysis was performed on the isolated compound **1**, and compound **1** was identified as citropten by comparing the analysis results with the previously reported literature [44].

Compound **1** (citropten): ^1^H NMR data (500 MHz, CDCl_3_) *δ*: 8.00 (d, *J* = 9.7 Hz, 1H, H-4), 6.40 (d, *J* = 1.7, 1H, H-8), 6.67 (d, *J* = 2.3, 1H, H-8), 6.27 (d, *J* = 2.0 Hz, 1H, H-6), 6.20 (d, *J* = 9.0 Hz, 1H, H-6), 6.15 (d, *J* = 9.2 Hz, 1H, H-3), 3.89 (s, 3H, OCH_3_), 3.73 (s, 3H, OCH_3_). ^13^C NMR data (500 MHz, CDCl_3_) *δ*: 163.8 (C-7), 161.7 (C-2), 157.0 (C-5), 156.9 (C-8a), 138.9 (C-4), 110.0 (C-3), 104.1 (C-4a), 94.9 (C-6), 92.9 (C-8), 56.0 (OCH_3_), 55.9 (OCH_3_).

### 4.5. Condition of Liquid Chromatography–Mass Spectrometry Analysis

Analyses were performed using a reversed-phase high-performance liquid chromatography (HPLC) system (Agilent model 1260 series, Santa Clara, CA, USA) with a Capcell pak C18 column (5 μm × 4.6 mm × 250 mm; Shiseido, Japan) and Agilent 6120 (Santa Clara, CA, USA) in the single-quadrupole positive ion mode. Chromatography was performed at room temperature at a flow rate of 1 mL/min, and 10 μL was analyzed for 50 min. The mobile phase consisted of 0.1% formic acid in water (A) and 0.1% formic acid in acetonitrile (B) in a ratio specified by the following binary gradient with linear interpolation: 0 min 5% B, 40 min 80% B and 50 min 5% B.

### 4.6. Reagents and Antibodies

Stimulatory antibodies against human CD3 and CD28 for the stimulation of T cells were obtained from BioXcell (West Lebanon, NH, USA). Recombinant human TNFα was purchased from PeproTech EC Ltd. (London, UK). MTT powder (1-(4,5-dimethylthiazol-2-yl)-3,5-diphenylformazan), TRIZOL reagent and radioimmunoprecipitation assay (RIPA) buffer for Western blot analysis, phorbol 12-myristate 13-acetate (PMA) and A23187 were provided by Sigma Chemical Co. (St. Louis, MO, USA). *Staphylococcus aureus* enterotoxin E (SEE) was obtained from Toxin Technology (Sarasota, FL, USA). ECL Western blotting detection reagents and an apoptosis AnnexinV/PI assay kit was purchased from Thermo Fisher Scientific (Waltham, MA, USA). SYBR Premix Ex Taq was provided by TaKaRa (Shiga, Japan). Anti-IL-2 antibodies, anti-β-actin and anti-ERK antibodies were obtained from Santa Cruz Biotechnology (Dallas, TX, USA). Antibodies against p65, LaminB, IκBα, phosphorylated IκBα, phosphorylated ERK, phosphorylated p38, p38, phosphorylated JNK and JNK were purchased from Cell Signaling Technology (Danvers, MA, USA). Anti-CD69 conjugated with APC were provided by eBiosciences. The RT PreMix kit was obtained from Enzynomics (Daejeon, Korea). DSS (molecular weight: 36,000–50,000 Da) was purchased from MP Biomedicals (Irvine, CA, USA).

### 4.7. Cell Confluency Check by IncuCyte Imaging System

Jurkat cells (1 × 10^4^/well) and HT-29 cells (1 × 10^4^/well) were treated with the indicated concentrations (0 to 40 μM) of citropten for 24 h; then, the cells were automatically marked in orange using the IncuCyte imaging system.

### 4.8. Cell Viability Check by MTT Assay

Cell viability was determined by performing an MTT assay. Jurkat cells (1 × 10^4^/well) and HT-29 cells (1 × 10^4^/well) were treated with the indicated concentrations (0 to 40 μM) of citropten for 24 h; then, MTT (500 μg/mL) was added for 2 h. After incubation, the supernatants were discarded, and formazan crystals at the bottom were dissolved with 200 μL of dimethyl sulfoxide (DMSO). To obtain the OD value, the plate was read at 540 nm. Cell viability was calculated using the obtained OD value and presented in % of control (0 μM).

### 4.9. AnnexinV/PI Apoptosis Assay

For the determination of apoptosis after treatment with citropten, an AnnexinV/PI apoptosis kit was used. Jurkat cells (5 × 10^5^/well) and HT-29 cells (5 × 10^5^/well) were treated with the indicated concentrations (0 to 40 μM) of citropten for 24 h and then stained with AnnexinV and PI following the manufacturer’s instructions. Cells were acquired through flow cytometry, and all single cells were gated by using BD software. AnnexinV and PI double-positive cells were obtained from double plots.

### 4.10. T Cell Stimulation

For T cell stimulation, three methods were used in the present study. For TCR-mediated stimulation, Jurkat T cells were replaced on the plate coated with anti-CD3 antibodies (20 μg/mL) and anti-CD28 soluble antibodies (7 μg/mL). For PMA/A23187 stimulation, Jurkat T cells were treated with 100 nM PMA and 1 μM A23187. Jurkat T cells were also stimulated by co-culturing them with same number of Raji B cells that were previously pulsed with SEE superantigen (1 µg/mL) for 1 h.

### 4.11. Determination of mRNA Levels by Real-Time Quantitative PCR

For the determination of mRNA levels via real-time quantitative PCR, harvested cells or colon tissues were lysed in TRIZOL reagents for total RNA isolation. The reverse transcription of the RNA was performed using an RT PreMix kit (Enzynomics, Daejeon, Korea). In addition, RNA was obtained after the colon tissue was washed with PBS 3 times to remove DSS. The primers used in the present study were as follows (forward and reverse primers): human *IL-2*, 5′-CAC GTC TTG CAC TTG TCA C-3′ and 5′-CCT TCT TGG GCA TGT AAA ACT-3′; human *TNFα*, 5′-CCT ACC AGA CCA AGG TCA AC-3′ and 5′-AGG GGG TAA TAA AGG GAT TG-3′; human *IL-1β*, 5′-GGA TAT GGA GCA ACA AGT GG-3′ and 5′-ATG TAC CAG TTG GGG AAC TG-3′; human *IL-8*, 5′-GTG CAG TTT TGC CAA GGA GT-3′ and 5′-TTA TGA ATT CTC AGC CCT CTT CAA AAA-3′; human *ICAM1*, 5′- AGC GGC TGA CGT GTG CAG TAA T-3′ and 5′-TCT GAG ACC TCT GGC TTC GTC A-3′; human *VCAM1*, 5′-GAT TCT GTG CCC ACA GTA AGG C-3′ and 5′-TGG TCA CAG AGC CAC CTT CTT G-3′; human *GAPDH*, 5′-CGG AGT CAA CGG ATT TGG TCG TAT-3′ and 5′-AGC CTT CTC CAT GGT GGT GAA GAC-3′; mouse *TNFα*, 5′-GGC AGG TCT ACT TTG GAG TCA TTG C-3′ and 5′-ACA TTC GAG GCT CCA GTG AAT TCG G-3′; mouse *IL-1β*, 5′-ATA ACC TGC TGG TGT GTG AC-3′ and 5′-AGG TGC TGA TGT ACC AGT TG-3′; mouse *IL-8*, 5′-ATG GCT GCT CAA GGC TGG TC-3′ and 5′-AGG CTT TTC ATG CTC AAC ACT AT-3′; mouse *Il-2*, 5′-TGA GCA GGA TGG AGA ATT ACA GG-3′ and 5′- GTC CAA GTT CAT CTT CTA GGC AC-3′; mouse *Ifng*, 5′-TCA AGT GGC ATA GAT GTG GAA GAA-3′ and 5′-TGG CTC TGC AGG ATT TTC ATG-3′; mouse *Il17*, 5′-TCC CCT CTG TCA TCT GGG AAG-3′ and 5′-CTC GAC CCT GAA AGT GAA GG-3′; mouse *GAPDH*, 5′–GCA CAG TCA AGG CCG AGA AT–3′ and 5′–GCC TTC TCC ATG GTG GTG AA–3′. PCR amplification was performed in a DNA Engine Opticon 1 continuous fluorescence detection system (MJ Research, Waltham, MA, USA) using SYBR Premix Ex Taq. It contained 1 μL of cDNA/control and gene-specific primers. Each PCR reaction was performed using the following conditions: 95 °C 30 s, 60 °C 30 s, 72 °C 30 s and plate read (detection of fluorescent product) for 40 cycles followed by 7 min of extension at 72 °C. Melting curve analysis was performed to characterize the dsDNA product by slowly raising the temperature (0.1 °C/s) from 60 °C to 95 °C with fluorescence data collected at 0.2 °C intervals. The mRNA levels of the genes were normalized to *GAPDH*. The gene expression was calculated using the following equation: Gene expression = 2^−^^ΔΔCT^, where ΔΔCT = (CT Target−CT *GAPDH*).

### 4.12. ELISA

For the detection of released IL-2 from activated Jurkat cells, ELISA was used following the manufacturer’s instructions (DuoSet^®^ ELISA kit, R&D Systems, Minneapolis, MN, USA).

### 4.13. Western Blot Analysis

For the detection of protein levels, Western blot analysis was performed. Harvested cells or colon tissues were lysed in RIPA buffer for 30 min on ice and centrifuged at 14,000 rpm for 20 min at 4 °C. For the separation of the nuclear extract, lysis was performed using NE-PER Nuclear and Cytoplasmic Extraction Reagents (Thermo fisher scientific, Waltham, MA, USA). Approximately 30 to 40 μg of the lysate was loaded for separation on 8–12% SDS–PAGE gels. Proteins were transferred onto PVDF membranes (Bio-Rad, Hercules, CA, USA), and membranes were blocked in 5% skim milk (1 h). After being rinsed, membranes were incubated with the indicated primary antibodies in TBS containing 0.1% Tween 20 (TBS-T) and 3% skim milk overnight. Excess primary antibodies were discarded by washing the membrane three times with TBS-T. The membranes were then incubated with 0.1 μg/mL peroxidase-labeled secondary antibodies (against rabbit or mouse) for 2 h. After three washes in TBS-T, bands were visualized with ECL Western blotting detection reagents (Thermo Fisher Scientific, Waltham, MA, USA) with an ImageQuant LAS 4000 (GE healthcare, Chicago, IL, USA). All detected bands were normalized with the intensity of the loading control proteins, and the ratio was calculated between experimental proteins and loading control proteins to be considered as 1 X. All normalized ratios were presented as fold changes compared to the ‘control’ group.

### 4.14. Determination of CD69 Expression by Flow Cytometry

For the detection of CD69 in activated Jurkat T cells, fluorescence was used after staining with flow cytometry. After stimulation, Jurkat T cells were stained with anti-CD69 antibodies conjugated with APC and acquired via flow cytometry. All live single cells were gated, and the mean fluorescence intensity was obtained by using BD flow cytometry software. Each mean fluorescence intensity is presented in a bar graph.

### 4.15. Induction of Colitis by Using DSS

The inflammatory colitis model was induced by feeding mice water containing DSS. Twenty mice were grouped into four groups, as follows: mice fed fresh water (water), mice fed water containing 2.5% DSS for seven days (DSS water), mice fed water containing 2.5% DSS and received oral administration of 10 mg/kg citropten every day for seven days (DSS water + CTP10) and mice fed water containing 2.5% DSS and received oral administration of 40 mg/kg citropten every day for seven days (DSS water + CTP40). Changes in body weight were examined for seven days.

### 4.16. Determination of Stool Scoring

Changes in the shape of stool were checked daily to examine the progress of inflammatory colitis as follows: 0 (normal), 2 (loose stool) and 4 (diarrhea).

### 4.17. Determination of Disease Activity Index

The disease activity index was monitored to evaluate the progression of inflammatory colitis according to the published criteria, and it involves the relative loss of body weight, shape of stool and bleeding on the stool and anus area. The scoring criteria were as follows: weight loss: 0 (no loss), 1 (1–5%), 2 (5–10%), 3 (10–20%) and 4 (more than 20%); stool form: 0 (normal), 2 (loose stool) and 4 (diarrhea); and bleeding: 0 (no blood), 1 (Hemoccult positive), 2 (Hemoccult positive and visual pellet bleeding) and 4 (bleeding around the anus).

### 4.18. H&E Staining and Determination of Histological Score

At day 7 post-induction of colitis, colon tissues were removed and prepared for histological analysis. The collected colons (0.5 cm) were fixed in 10% paraformaldehyde and embedded using paraffin. Embedded tissues were cut (5-μm-thick axial sections) and put on slide glass to be deparaffinized. Deparaffinized tissues were stained with H&E for histological analysis. The histological scores were evaluated according to previously reported methods [45].

### 4.19. Statistics

The mean values ± SD were calculated from the data collected from three independent experiments performed on separate days and are presented in bar graphs. For mice experiments, the mean values ± SD were calculated from the data obtained from five mice experiments and are presented in bar or dot graphs. One-way ANOVA was used to determine significance (*p* value), and Tukey’s post hoc test was used after one-way ANOVA. * indicates differences between the two indicated groups considered significant at *p* < 0.05.

## Data Availability

Not applicable.
